# The effects of phenylalanine on exercise-induced fat oxidation: a preliminary, double-blind, placebo-controlled, crossover trial

**DOI:** 10.1186/s12970-017-0191-x

**Published:** 2017-09-12

**Authors:** Keisuke Ueda, Chiaki Sanbongi, Makoto Yamaguchi, Shuji Ikegami, Takafumi Hamaoka, Satoshi Fujita

**Affiliations:** 1 Food Science Research Labs, R&D Division, Meiji Co., Ltd., 540 Naruda, Odawara, Kanagawa 250-0862 Japan; 2 College of Sport and Health Science, Ritsumeikan University, 1-1-1 Nojihigashi, Kusatsu-shi, Shiga 525-8577 Japan; 3 Department of Sports Medicine for Health Promotion, Tokyo Medical University, 6-1-1 Shinjuku, Shinjuku-ku, Tokyo, 160-8402 Japan

**Keywords:** Pre-exercise nutrition, Amino acid, Metabolism, Hormones

## Abstract

**Background:**

When combined with exercise, dietary amino acid (AA) supplementation is an effective method for accelerating fat mobilization. However, the effects of single AAs combined with exercise on fat oxidation remains unclear. We hypothesized that consumption of a specific amino acid, L- phenylalanine, may result in the secretion of glucagon, and when combined with exercise may promote fat oxidation.

**Methods:**

Six healthy, active male volunteers were randomized in a crossover study to ingest either phenylalanine (3 g/dose) or placebo. Thirty minutes after ingestion each subject performed workload trials on a cycle ergometer for 1 h at 50% of maximal oxygen consumption.

**Results:**

Oral intake of phenylalanine caused a significant increase in the concentrations of plasma glycerol and glucagon during exercise. The respiratory exchange ratio was also decreased significantly following ingestion of phenylalanine.

**Conclusion:**

These results suggested that pre-exercise supplementation of phenylalanine may stimulate whole body fat oxidation. No serious or study-related adverse events were observed.

**Trial registration:**

UMIN000027502 Registered 26 May 2017. Restrospectively registered.

## Background

Regular exercise is an important strategy to implement to help prevent obesity [1]. According to the exercise prescription recommended in the American College of Sports Medicine [2] guidelines, 45–60 min of exercise should be targeted to ensure sufficient energy expenditure in obese people. In particular, the blood levels of several hormones including catecholamines, glucagon, growth hormone, and cortisol are increased during exercise compared with levels in rest periods [3]. Moreover, several studies reported on acute exercise and lipid utilization, and related hormones in human [4–6]. Therefore, we hypothesize that a combination of exercise and factors that affect the secretion of some of these hormones may be effective for increasing fat catabolism and energy expenditure.

When combined with exercise, dietary amino acid (AA) supplementation is an effective method for accelerating fat mobilization [7, 8]. It has been reported that previously that pre-ingestion of a single dose of a mixture of specific amino acids (AAs) enhanced lipolysis and hepatic ketogenesis during and after exercise by stimulating glucagon secretion [9, 10]. However, the effects of single AAs combined with exercise on fat oxidation remains unclear. L-phenylalanine (Phe), a tyrosine precursor, is an essential amino acid, and is a substrate for tyrosine hydroxylase, the enzyme that catalyzes the rate-limiting step in catecholamine synthesis [11]. It is known that Phe is a dietary requirement for protein synthesis. Nuttall et al. showed that a single oral administration of a large amount of Phe (1 mmol/kg lean body mass) acted as a nutrient-signaling molecule that stimulated an increase in insulin and glucagon concentration, and regulated glucose metabolism [12]. However, whether or not ingestion of small amounts of Phe combined with or without exercise has similar effects has yet to be determined. Therefore, the purpose of this randomized, double-blind, placebo-controlled, crossover trial was to investigate hormone secretion and substrate catabolism induced by Phe during exercise.

## Methods

### Trial design

This was a randomized, double-blind, placebo-controlled crossover study, conducted at Ritsumeikan University at Shiga, Japan. The study protocol was approved by the Ethics Committee for Human Experiments at Ritsumeikan University and the Meiji Institutional Review Board. All study participants provided written informed consent prior to participation in the study. The study was performed in accordance with the ethical standards of the 1964 Declaration of Helsinki and its later amendments. The study protocol was registered in the UMIN Clinical Trials Registry (UMIN000027502) on May 26, 2017 (restrospectively registered).

### Subjects

The inclusion criteria were healthy young men aged 20 to 40 years old. Exclusion criteria consisted of individuals with a history or current condition of severe disease (such as liver disorder, cardiovascular disorder, respiratory disorder, renal disorder, and hypertension), anemia, and those who were judged ineligible by the study physician due to medical examination consultation history or other reasons. Six healthy, active young men were recruited as study volunteers. The baseline characteristics (mean ± deviation (SD)) of the participants were: age, 23.7 ± 1.0 yr.; height, 172.6 ± 7.9 cm; body mass, 67.7 ± 4.9 kg; body mass index (BMI), 22.7 ± 1.1 kg/m^2^; and maximum oxygen uptake, 52.4 ± 4.2 mL/min/kg.

### Experimental procedures

The study involved three visits to the laboratory. At the first visit, maximal oxygen uptake *VO*_2max_ (mL/kg/min) and maximal heart rate (HR) (beats/min) were measured using an incremental cycle exercise test on a cycle ergometer (828E Monark cycle ergometer). The incremental cycle exercise began at a work rate of 60 W (30–90 W) with power output being increased in 15 W·min^−1^ steps until the subject could not maintain a fixed pedaling frequency of 60 rpm. The subjects were encouraged to exercise at maximum intensity during the ergometer test. Heart rate and rating of perceived exertion (RPE) were monitored every minute during exercise. RPE was obtained using the modified Borg scale. VO_2_ was monitored by breath–by–breath assessment using a respiratory gas analyzer (Aeromonitor AE-310SRD, Minato Medical Science Co., Ltd., Osaka, Japan). The highest 30-s averaged value of VO_2_ during the exercise test was designated as VO_2_peak if three of the following four criteria were met: (1) plateau in VO_2_ with an increase in external work, (2) maximal respiratory exchange ratio ≥ 1.1, (3) HR ≥ 200 beats/min. The results of exhaustion testing were used to calculate the power output equivalent to 50% *VO*_2max._ The remaining two visits were separated by at least six days. Dietary intake was self-recorded by the subjects during the study period. The subjects were instructed to refrain from binge eating, strenuous exercise, or drinking alcohol for 24 h prior to each trial and were also instructed to sleep more than eight hours the evening before each visit. At approximately 21:00 on the day before the second and third visits the subjects consumed the same meals that contained 694 kcal (carbohydrate:fat:protein ratio; 57:28:15). The subjects had no food or drink except water between the last meal and the start of each trial. Individual trials were performed at a similar time of the day for each subject (±3 h) to avoid any influence of circadian rhythm on the results.

**Fig. 1:**
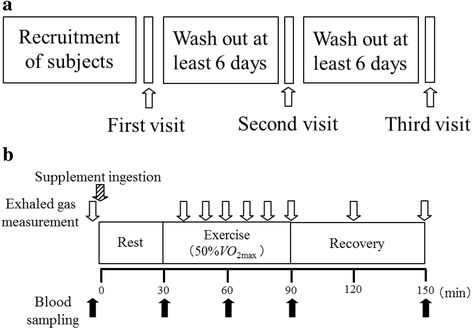
Study design: Study schedule **a** and schedule on experimental trial days (Visit 2 and Visit 3; **b**)

During the second and third visits the subjects participated in the main experimental trials. Blood samples were drawn from the antecubital vein. The subjects were then randomized to ingest 150 mL of ordinary tap water and either a cellulose capsule containing 3 g of Phe (Kyowa Hakko Bio Co, Ltd., Tokyo, Japan) as the active sample or an empty cellulose capsule (Matsutani Chemical Industry Co., Ltd., Hyogo, Japan) as the placebo (designated as 0 min). The treatments were switched at the crossover phase of the study. After sitting for 30 min (rest period), the subjects mounted a cycle ergometer and commenced cycling for 60 min at a constant power output equivalent to 50% *VO*_2max_ (exercise period). After exercising, the subjects rested for 60 min in the supine position (post-exercise period). Blood samples were collected at before ingestion of test sample, and 30, 60, 90, and 150 minutes after ingestion. HR was recorded and exhaled air samples were collected throughout the rest, exercise, and post-exercise phases. The tests were conducted in a quiet environment in a controlled room at a temperature of 21 ± 2 °C and humidity of 45 ± 5%. The study design is summarized in Fig. 1.

### Exhaled gas analysis

Exhaled oxygen and carbon dioxide concentrations were measured by the breath-by-breath method using a same respiration metabolism monitor system de scribed above. Respiratory exchange ratio (RER) was calculated using the expiratory gas measurements 30 s before and after every 10 min period during exercise and every 30 min during recovery.

### Blood sampling

Whole blood was collected in a vacutainer containing sodium fluoride and ethylenediaminetetraacetic acid (EDTA)-2Na and stored at 4 °C for later analysis of glucose and lactate concentrations. Whole blood from a EDTA-2Na vacutainer with added aprotinin was centrifuged immediately at 1200 g for 10 min at 4 °C, and the plasma separated and frozen immediately at −80 °C for later analysis of glucagon concentration. Whole blood from a EDTA-2Na vacutainer without added aprotinin was centrifuged immediately at 1200 g for 10 min at 4 °C, and the plasma stored at 4 °C for analysis of cortisol concentration. Whole blood from a plain vacutainer was allowed to stand at room temperature for 20 min and then centrifuged at 1200 g for 10 min at 4^o^ C, followed by separation of the serum into two vials. One vial was stored at 4^o^ C for analysis of FFA and growth hormone concentrations, while the other vial was frozen at -80^o^ C for later analysis of acetoacetic acid, 3-hydroxybutyrate, and glycerol concentrations.

Plasma glucagon concentration was measured using the quantitative sandwich enzyme immunoassay (Glucagon Immunoassay R&D systems, Minnesota, USA). Plasma glucose and lactate values were measured using the YSI 2300 STAT Plus Glucose & Lactate Analyzer (YSI Inc., Yellow Springs, Ohio, USA). Serum growth hormone (Access hGH, Beckman Coulter, Inc., USA) and plasma cortisol (Access cortisol, Beckman Coulter, Inc., USA) concentrations were measured by a chemiluminescent enzyme immunoassay. And serum acetoacetic acid, 3-hydroxybutyrate (Total ketone bodies Kainos, 3-HB Kainos, Kainos Co., Ltd., Japan), FFAs (NEFA-HR, Wako Pure Chemical Industries Ltd., Osaka, Japan), and glycerol (Glycerol Colorimetric Assay Kit, Cayman Chemical, Ann Arbor, MI, USA) concentrations were measured by enzymatic methods. The assays to measure acetoacetic acid, 3-hydroxybutyrate, FFAs were performed at Medic Co., Ltd. (Shiga, Japan), and the other assays at the laboratory of Ritsumeikan University.

### Statistical analysis

Data were expressed as mean ± SD and analyzed using Microsoft Excel (Microsoft Corp., Redmond, WA, USA). All variables were tested for normal distribution by the F-test using StatView-J 5.0 software (Abacus Conceps, Berkeley, CA, USA). If the data were normally distributed, repeated measures two-factor analysis of variance (ANOVA, time-treatment) was used to examine differences between the biochemical parameters from the two trials. Moreover, when the ANOVA revealed significant effects or interactions between factors, Tukey’s post-hoc test was used to detect significant differences between the two treatments. On the other hand, if the data were skewed distribution, Freidman test was used to examine differences. Statistical significance was set at *P* values <0.05.

## Results

### Cardiorespiratory responses

In RER, there were no outliers, and the data were normally distributed. The changes in RER are shown in Table 1. ANOVA showed a significant treatment × time interaction for RER (treatment *P* = 0.286; time *P* < 0.01; interaction *P* < 0.01), with Tukey’s post-hoc test showing significant differences between treatments at 40 and 120 min (*P* < 0.05).

**Table 1:** Cardiorespiratory responses of participants

	0 min		40 min		50 min		60 min		70 min		80 min		90 min		120 min		150 min	
	Phe	Placebo	Phe	Placebo	Phe	Placebo	Phe	Placebo	Phe	Placebo	Phe	Placebo	Phe	Placebo	Phe	Placebo	Phe	Placebo
RER	0.83 ± 0.030	0.85 ± 0.041	0.90 ± 0.063*	0.97 ± 0.042	0.96 ± 0.020	0.94 ± 0.036	0.95 ± 0.018	0.93 ± 0.045	0.94 ± 0.019	0.93 ± 0.022	0.93 ± 0.026	0.93 ± 0.027	0.93 ± 0.022	0.93 ± 0.016	0.74 ± 0.057*	0.86 ± 0.048	0.80 ± 0.039	0.80 ± 0.034
F-value	1.2		10		0.50		0.48		0.20		0.00		0.030		29		0.078	

Values are mean ± SD (*n* = 6)

*p value for the two factor analysis of variance and Tukey’s post-hoc test if data were normally distributed, and Friedman test if not

### Biochemical parameters

In blood glycerol, FFA, glucose, and ketone bodies, there were no outliers, and the data were normally distributed. However, in blood lactate, there were outliers, and the data were not normally distributed. The biochemical parameters of the two treatments are summarized in Table 2. ANOVA showed a significant treatment × time interaction for plasma serum glycerol concentration (treatment *P* = 0.022; time *P* < 0.01; interaction *P* < 0.01), with Tukey’s post-hoc test revealing significant differences between treatments at 90 min (*P* < 0.05). The blood concentrations of glucose, lactate, FFA, and ketone bodies were not significantly different between the two treatments throughout the experimental period.

**Table 2:** Effects of phenylalanine n the blood biochemical parameters

	0 min		30 min		60 min		90 min		150 min		Two factor ANOVA *P* value		
	Phe	Placebo	Phe	Placebo	Phe	Placebo	Phe	Placebo	Phe	Placebo	time	treatment	interaction
Glycerol (mg/L)	3.34 ± 1.55	3.84 ± 1.60	3.24 ± 1.38	3.48 ± 1.77	5.34 ± 0.82	4.83 ± 1.51	15.15 ± 3.02^*^	7.30 ± 1.93	4.06 ± 1.59	3.64 ± 1.18	*p* < 0.01	0.022	*p* < 0.01
F-value	0.25		0.056		0.27		62		0.18				
FFAs (mEq/L)	0.58 ± 0.16	0.50 ± 0.21	0.55 ± 0.17	0.44 ± 0.15	0.39 ± 0.11	0.42 ± 0.20	0.69 ± 0.28	0.64 ± 0.33	0.79 ± 0.30	0.74 ± 0.17	*p* < 0.01	0.65	0.67
F-value	0.35		0.82		0.059		0.15		0.16				
Total ketone bodies (μmol/L)	120 ± 109	113 ± 143	160 ± 143	92.2 ± 97.5	114 ± 50.4	60.3 ± 24.1	169 ± 86.0	121 ± 107	387 ± 401	333 ± 412	*p* < 0.01	0.62	0.99
F-value	0.0043		0.33		0.21		0.17		0.21				
Glucose (nmol/L)	5.08 ± 0.48	5.18 ± 0.35	5.21 ± 0.53	5.09 ± 0.67	4.89 ± 0.56	4.87 ± 0.63	5.35 ± 0.83	4.89 ± 0.51	5.15 ± 0.56	5.04 ± 0.66	*p* < 0.01	0.69	0.32
F-value	0.075		0.12		0.0029		1.8		0.10				
Lactate (nmol/L)	1.17 ± 0.22	1.18 ± 0.18	1.24 ± 0.18	1.07 ± 0.31	3.72 ± 1.81	3.49 ± 1.05	3.46 ± 2.59	2.70 ± 0.83	1.13 ± 0.14	1.12 ± 0.15	–	–	–

Values are mean ± SD (n = 6). FFA = free fatty acids

*p value for the two factor analysis of variance and Tukey’s post-hoc test if data were normally distributed, and Friedman test if not

### Circulating hormones

In blood glucagon and cortisol, there were no outliers, and the data were normally distributed. However, in blood lactate, there were outliers, and the data were not normally distributed. The concentrations of circulating hormones during each treatment are shown in Table 3. ANOVA showed a significant treatment × time interaction for plasma glucagon (treatment *P* = 0.025; time *P* = 0.035; interaction *P* = 0.039), with Tukey’s post-hoc test showing significant differences between treatments at 30, 60, 90, and 150 min (*P* < 0.05). Growth hormone and cortisol concentrations did not differ significantly between the two treatments throughout the experimental period.

**Table 3:** Effects of phenylalanine on the circulating hormones

	0 min		30 min		60 min		90 min		150 min		Two factor ANOVA		
											P value		
	Phe	Placebo	Phe	Placebo	Phe	Placebo	Phe	Placebo	Phe	Placebo	time	treatment	interaction
Glucagon (ng/L)	62.9 ± 7.81	66.0 ± 19.1	90.1 ± 29.1^*^	63.6 ± 21.8	73.5 ± 18.6^*^	52.3 ± 8.78	92.2 ± 18.8^*^	61.0 ± 7.78	92.3 ± 27.2^*^	61.4 ± 19.7	0.04	0.03	0.04
F value	0.078		5.7		3.6		7.8		7.7				
Cortisol (μg/L)	106 ± 52.2	113 ± 32.7	109 ± 41.4	77.6± 23.7	146 ± 53.2	91.9± 34.9	173 ± 70.9	122 ± 27.5	157 ± 51.3	89.5 ± 31.3	*p* < 0.01	0.05	0.11
F value	0.072		1.5		4.4		4.0		7.0				
Growth hormone (μg/L)	1.30 ± 2.01	0.14 ± 0.08	1.44 ± 2.80	0.30 ± 0.46	9.72 ± 6.86	13.1 ± 5.61	13.4 ± 14.2	14.2 ± 8.5	1.51 ± 0.72	1.13 ± 1.12	–	–	–

Values are mean ± SD (n = 6)

*p value for the two factor analysis of variance and Tukey’s post-hoc test if data were normally distributed, and Friedman test if not

## Discussion

This study in healthy active young men investigated the acute effects of Phe supplementation combined with exercise on hormone secretion and substrate oxidation. The study showed that, compared with ingestion of a placebo, ingestion of the Phe supplement significantly increased the concentrations of glycerol and glucagon. The RER was also decreased significantly by Phe ingestion. These findings suggest that whole body lipid oxidation increased and that pre-exercise supplementation of Phe stimulated fat oxidation. To the author’s knowledge this is the first study to examine the effects of Phe on human fat oxidation combined with exercise. Although considerable evidence exists on the safety of Phe consumption in humans [13] there is no evidence on the functional effects related to fat oxidation combined with exercise. The data presented in this study lay the groundwork for further investigations on Phe supplementation in sport.

The main mechanism for the stimulation of fat oxidation following Phe administration may be via glucagon secretion. Glucagon is a key hormone involved in fat catabolism during exercise [14, 15]. Previous reports have noted that several widely divergent effect of glucagon appear to be mediated by a effector, adenosine 3*′*,5*′*-cyclic monophosphate (cAMP) [16]. The best-known mechanism mediating lipolysis is the cAMP pathway, wherein increased levels of cAMP activate cAMP-dependent protein kinase A (PKA). In this pathway, hormone sensitive lipase (HSL) is phosphorylated by PKA and then translocates from the cytoplasm to the lipid droplet surface, where it interacts with perilipin A, the result of which is a subsequent release of free fatty acids [17, 18]. HSL is the most important lipase in lipolysis and is subject to hormonal regulation [19]. In addition to HSL, adipose triglyceride lipase is expressed predominantly in adipose tissue and is considered the rate-limiting lipolytic enzyme in adipocytes [20, 21]. Therefore, an additional effect by Phe supplementation combined with exercise may stimulate the cAMP-dependent cascade pathway. Previous studies have suggested that glucagon secretion is regulated by gut hormones including glicentin, GLP-1, GIP, and GLP-2 and other peptides secreted by the gastrointestinal tract such as gastrin-releasing peptide (GRP), cholecystokinin (CCK), and secretin [22]. Further studies are necessary to determine whether or not these hormones are involved in the stimulation of fat oxidation caused by Phe administration.

It has been reported that a single pre-ingestion dose of mixtures of either 17 specific AAs [9] or 3 AAs [10] enhanced lipolysis and hepatic ketogenesis during and after exercise by stimulating glucagon secretion. However, in the current study pre-exercise ingestion of Phe increased glycerol concentrations but not ketone body levels. The results suggest that pre-exercise ingestion of Phe markedly stimulate lipolysis but not hepatic ketogenesis. Taken together, these results indicate that ingestion of a specific AA stimulates fat oxidation efficiently during exercise.

In 1970 Harper et al. [23] published a review of the effects of disproportionate levels of AA intake, with the information subsequently being updated in 1984 by Benevenga and Steeles [24]. There is concern that Phe supplementation may be associated with abnormal brain development known to occur in humans with phenylketonuria, a condition that results in a buildup of Phe and its metabolites in the blood [25]. However, no adverse effects were noted in humans given either a single oral dose of up to 10 g [26], ~30 g i.v. [27], or 3–4 g orally as aspartame [26]. No serious or study-related adverse events were observed in the current trial and therefore we conclude that Phe supplementation may be a safe method for accelerating fat oxidation during exercise.

This study has several strengths worth mentioning. First, the trial was a placebo-controlled, double-blind, crossover, randomized, controlled design and therefore the findings are highly reliable. Second, evaluation of the respiratory exchange ratio is regarded as the gold standard for evaluating whole body fat oxidation. Third, the study only administered 3 g of Phe prior to exercise, a low-dose of amino acid supplementation that could be used easily elsewhere.

In contrast, we must also note some limitations. First, the number of enrolled participants was only six of young male. Therefore, the data robustness may not be high. Second, we failed to measure blood insulin levels. Insulin signaling is a key factor for glucagon secretion [12], so further studies are needed to demonstrate the effect of Phe combined with exercise for insulin secretion. However, stimulation of sympathetic nervous system by exercise suppresses insulin secretion [3]. Moreover, in our previous report, pre-ingestion of the amino acid mixture containing 1.5 g of Phe did not stimulate insulin secretion during 50% VO_2_max exercise [10]. Therefore, we thought that the influence of insulin secretion on this fat oxidation effect mediated by Phe ingestion combined with exercise is small. Third, we did not present the data of energy expenditure, so it remains unclear this higher fat oxidation leads to obesity prevention or weight control. However, ingestion of Phe may increase fat mobilization, and this effect might to lead, at least in part, to an increase in fat oxidation during exercise. Further studies are needed to investigate whether this acute response induced by the administration of Phe is sustained if the supplement is ingested over several weeks.

## Conclusions

In conclusion, pre-exercise ingestion of a Phe supplement significantly accelerated secretion of glucagon during both rest and exercise. Furthermore especially serum glycerol levels increased significantly during exercise, indicating a shift towards fat oxidation.

## References

[1] Jakicic JM (2003). Exercise in the treatment of obesity. Endocrinol Metab Clin N Am.

[2] Whaley MH, American College of Sports Medicine (2006). Clinical Ecercise Testing. ACSM’s Guidelines for Exercise Testing and Prescription.

[3] Richter EA, Kiens B, Mizuno M, Strange S (1989). Insulin action in human thighs after one-legged immobilization. J Appl Physiol.

[4] Numao S, Hayashi Y, Katayama Y, Matsuo T, Tomita T, Ohkawara K, Nakata Y, Tanaka K (2006). Effects of obesity phenotype on fat metabolism in obese men during endurance exercise. Int J Obes.

[5] Miyashita M (2008). Effects of continuous versus accumulated activity patterns on postprandial triacylglycerol concentrations in obese men. Int J Obes.

[6] Saunders TJ, Palombella A, McGuire KA, Janiszewski PM, Després JP, Ross R. Acute exercise increases adiponectin levels in abdominally obese men. J Nutr Metab. 2012;2012:148729. 10.1155/2012/148729 PMC3369484 22701167

[7] Lucotti P, Setola E, Monti LD, Galluccio E, Costa S, Sandoli EP, Fermo I, Rabaiotti G, Gatti R, Piatti P (2006). Beneficial effects of a long-term oral L-arginine treatment added to a hypocaloric diet and exercise training program in obese, insulin-resistant type 2 diabetic patients. Am J Physiol Endocrinol Metab.

[8] Forbes SC, Harber V, Bell GJ (2013). The acute effects of L-arginine on hormonal and metabolic responses during submaximal exercise in trained cyclists. Int J Sport Nutr Exerc Metab.

[9] Ueda K, Nakamura Y, Yamaguchi M, Mori T, Uchida M, Fujita S (2016). Amino Acid Mixture Enriched with Arginine, Alanine, and Phenylalanine Stimulates Fat Metabolism During Exercise. Int J Sport Nutr Exerc Metab.

[10] Ueda K, Sanbongi C, Takai S, Ikegami S, Fujita S (2017). Combination of aerobic exercise and an arginine, alanine, and phenylalanine mixture increases fat mobilization and ketone body synthesis. Biosci Biotechnol Biochem.

[11] Fernstrom JD, Fernstrom MH (2007). Tyrosine, phenylalanine, and catecholamine synthesis and function in the brain. J Nutr.

[12] Nuttall FQ, Schwein KJ, Gannon MC (2006). Effect of orally administered phenylalanine with and ithout glucose on insulin, glucagon and glucose concentrations. Horm Metab Res.

[13] Prensky AL, Fishman MA, Daftari B (1974). Recovery of rat brain from a brief hyperphenylalaninemic insult early in development. Brain Res.

[14] Wasserman DH, Spalding JA, Lacy DB, Colburn CA, Goldstein RE, Cherringon AD (1989). Importance of intrahepatic mechanisms to gluconeogenesis from alanine during exercise and recovery. Am J Physiol.

[15] Wolfe RR, Nadel ER, Shaw JHF, Stephenson LA, Wolfe MH (1986). Role of changes in insulin and glucagon in glucose homeostasis in exercise. J Clin Invest.

[16] Blecher M, Merlino NS, Ro'Ane JT, Flynn PD (1969). Independence of the effects of epinephrine, glucagon, and adrenocorticotropin on glucose utilization from those on lipolysis in isolated rat adipose cells. J Biol Chem.

[17] Holm C (2003). Molecular mechanisms regulating hormone-sensitive lipase and lipolysis. Biochem Soc Trans.

[18] Carmen GY, Victor SM (2006). Signalling mechanisms regulating lipolysis. Cell Signal.

[19] Rayalam S, Della-Fera MA, Baile CA (2008). Phytochemicals and regulation of the adipocyte life cycle. J Nutr Biochem.

[20] Haemmerle G, Lass A, Zimmermann R (2006). Defective lipolysis and altered energy metabolism in mice lacking adipose triglyceride lipase. Science.

[21] Zechner R, Strauss JG, Haemmerle G, Lass A, Zimmermann R (2005). Lipolysis: pathway under construction. Curr Opin Lipidol.

[22] Holst JJ (2011). Regulation of Glucagon Secretion by Incretins. Diabetes Obes Metab.

[23] Harper AE, Benevenga NJ, Wohlheuter RM (1970). Effects of ingestion of disproportionate amounts of amino acids. Physiol Rev.

[24] Benevenga NJ, Steele RD (1984). Adverse effects of excessive consumption of amino acids. Annu Rev Nutr.

[25] Garlick PJ. The Nature of Human Hazards Associated with Excessive Intake of Amino Acids. J Nutr. 2004;134:1633S–9S. 10.1093/jn/134.6.1633S 15173443

[26] Ryan-Harshman M, Leiter LA, Anderson GH (1987). Phenylalanine and aspartame fail to alter feeding behavior, mood and arousal in men. Physiol Behav.

[27] Floyd JC, Fajans SS, Conn JWA, Knopf RF, Rull J (1966). Stimulation of insulin secretion by amino acids. J Clin Invest.

